# Raising the contraceptive prevalence rate to 50% by 2025 in Pakistan: an analysis of number of users and service delivery channels

**DOI:** 10.1186/s12961-022-00950-y

**Published:** 2023-01-12

**Authors:** Mujahid Abdullah, Faiq Bilal, Romesa Khan, Azadeh Ahmed, Aamir Ashraf Khawaja, Faisal Sultan, Adnan Ahmad Khan

**Affiliations:** 1 Akhter Hameed Khan Foundation, Islamabad, Pakistan; 2 Research and Development Solutions, Islamabad, Pakistan; 3 Ministry of National Health Services, Regulation and Coordination, Islamabad, Pakistan; 4 Shaukat Khanum Memorial Cancer Hospital & Research Centre, Lahore, Pakistan

**Keywords:** Family planning programming, Data triangulation, Population metrics, Public service delivery

## Abstract

**Background:**

Pakistan is the fifth most populous country in the world, with a population that is growing at 2.4% annually. Despite considerable political will, including a national commitment that was endorsed by the president to raise the contraceptive prevalence rate (CPR) to 50% by 2025, it has stagnated at around 30–35%. Much of the dialogue on raising CPR is hypothetical and revolves around percentage point change rather than an actual number of women that must be served.

**Methods:**

The Demographic and Health Survey 2017–18 (DHS 2017–18) provides information about the channels through which users receive family planning (FP) services and disaggregates this information at the provincial level. Proportions of users from each of these channels were multiplied by the Pakistan Census-2017 populations to arrive at the number of users. These users were compared with the total FP users and the number of women that had used any FP service in the past 12 months. Linear estimations of population were applied to calculate population numbers in 2025.

**Results:**

The national target of 50% CPR by 2025 translates to a population of 20.02 million users. Currently, 11.26 million married women of reproductive age (MWRA) use any method, 8.22 million use a modern method and 4.94 million received this service in the past 12 months. Of these, 2.7 million did so from social marketing outlets, 0.76 million from public sector outreach through lady health workers (LHWs), 0.55 million from private sector and 0.88 million from public sector facilities. However, arriving at the CPR target means expanding annual service delivery from 4.94 to 13.7 million users. Since social marketing and LHW outreach may have become saturated, only public and private health facilities are the likely channels for such an expansion.

**Conclusions:**

We demonstrate triangulation of the survey data with the census data as a simple policy analysis tool that can help decision-makers estimate the quantum of services they must provide. Such an analysis also allows an understanding of the utilization patterns of each of these channels. In Pakistan’s context, underutilization of funds and existing facilities suggests that increased funding or more providers will likely not be helpful. The policy changes that will likely be most effective include adding outreach to support existing public and private sector facilities while ensuring that procurement of commodities is prioritized.

## Background

The global population is starting to stabilize after rapid expansion in the past two centuries [1, 2]. However, fertility rates remain high for many developing countries, where the population growth at times outpaces any gains in economic development, education and health, impacts the quality of life by reducing access to these essential services and even leads to violence and extremism [3, 4].

Pakistan is one such example. It is the world’s fifth most populous country, and its current population growth rate of 2.4% sets it on a path where its population will increase from the current 208 to 310 million by 2050 [5, 6]. This will profoundly impact Pakistan’s socioeconomic status and environment, and affect its ability to achieve its Sustainable Development Goals (SDGs), especially SDG 3 (good health and well-being) [7].

To address this rapid population growth, Pakistan committed at the London Summit on Family Planning 2012 to raise its contraceptive prevalence rate (CPR) to 55% by 2020 [8]. This commitment has since been institutionalized nationally in the recommendations of the Council of Common Interests (CCI) and revised at 50% by 2025 [9]. However, despite political commitment, allocated budgets and large-scale family planning (FP) programmes, Pakistan’s CPR has remained stagnant in the 30–35% range since 2007 [9–12].

The current debate on improving CPR in Pakistan revolves around percentage point changes rather than the actual number of users that must be served. This hides the quantum of changes needed, along with the specificity of what services are needed, in which quantities and at what locations. The numbers-based approach helps us quantify the actual number of users needed to reach 50% CPR, which is directly linked with service delivery channels that help us determine number of users per channel. Therefore, representing quantity of users needed provides a more precise picture for strategy development of FP programming and population policy-making.

We use data from the Population Census 2017 of Pakistan and the Pakistan Demographic and Health Survey (DHS) 2017–18 to outline the abovementioned argument. We also propose to initiate a secondary discussion on the relative roles and additional number of users required for the four different service delivery channels, such as public and private facilities, social marketing (pharmacies, markets, shops, etc.) and public sector outreach through lady health workers (LHWs) who increase awareness about FP services through door-to-door visits. FP services are provided free of cost in public facilities and through LHWs; however, these services are self-funded at private facilities and social marketing sites [13, 14].

## Methods

We used data on modern FP method users from the Woman’s Questionnaire of the Pakistan DHS 2017–18. Modern methods are classified as short-term and long-term methods. Short-term methods include male condoms, contraceptive pills, and lactation amenorrhea methods, whereas long-term methods include male and female sterilization, intrauterine contraceptive devices (IUCDs), implants and ligation. DHS data are used to understand the various channels from which married women of reproductive age (MWRA) receive their contraceptives and services, disaggregated by province (Punjab, Sindh, Khyber Pakhtunkhwa [KP], Balochistan and Islamabad). All our national analyses excluded the autonomous regions of Azad Jammu and Kashmir (AJK) and Gilgit-Baltistan (GB).

Since DHS uses a stratified two-stage random sampling design, we incorporated design effects in our analyses and used the provided sampling weights such that our sample was representative of populations in each province [10]. We also took CPR targets for 2025 at the provincial and national levels as recommended by CCI [15], which are given as Punjab (54%), Sindh (47%), KP (46%), Balochistan (36%), Islamabad (62%) and national (50%). We took population numbers of MWRA aged 15–49 years for all provinces from the Population Census-2017 of Pakistan [16].

First, we cross-tabulated weighted sources of modern FP methods and types of methods currently being used at national levels and across provinces. Then we integrated and converted these sources of modern FP methods from DHS into four main channels: public facility, private facility, public sector outreach through LHWs and social marketing (Appendix, Table 3). That gave us a percentage of users currently availing themselves of modern FP resources through these four channels. Those percentages were then multiplied by the total population of MWRA to estimate the actual number of users being served through these channels. Then we calculated the number of MWRA that were served in the past 12 months, by excluding those current users, for example, who had received an IUCD or sterilization surgery in a previous year; they would continue to be counted among modern method users, albeit not a user of the service in the past 12 months.

We took current population growth rates of Pakistan (2.4%), Punjab (2.1%), Sindh (2.4%), KP (2.8%), Balochistan (3.3%) and Islamabad (4.8%) [15, 17], and applied linear growth assumptions to estimate total MWRA populations in 2025. We then estimated the number of additional users that must be reached by 2025 to achieve 50% CPR nationally, by excluding the number of users receiving FP services each year from the total estimated MWRA population that must be reached. We assumed that the total number of traditional method users may not change drastically by 2025 from its current number [10]. Binomial exact 95% confidence intervals were then calculated for these estimates. Finally, we calculated the percentage increase required to reach the national goal of 50% CPR by 2025, through all channels combined, and then through public and private facilities only. Then we converted the percentage increase to actual numbers to represent how many more MWRA users were needed to reach 50% CPR.

## Results

The national CPR of 34.2% and 24.3% for modern methods translates into 11.3 million women who use any FP method and 8.2 million who use a modern method, respectively. Among these, 4.9 million had received FP services within the past 12 months, and therefore represent the total annual output of the FP service delivery system in Pakistan.

The 4.9 million women who currently use services for a modern method each year do so from one of the four primary channels. These include social marketing for self-procured short-term contraceptives from a store or pharmacy (2.702 million users), government outreach through LHWs (0.763 million users), and a public (0.877 million) or a private (0.553 million) healthcare provider or clinic (Table 1).

**Table 1:** Channels of modern FP methods and number of MWRA users (in millions)

	Punjab	Sindh	KP	Balochistan	Islamabad	National
Public facility	0.441 [0.367, 0.500]	0.269 [0.232, 0.304]	0.108 [0.084, 0.134]	0.041 [0.030, 0.052]	0.015 [0.012, 0.019]	0.877 [0.821, 0.963]
Private facility	0.264 [0.218, 0.327]	0.122 [0.102, 0.156]	0.128 [0.102, 0.155]	0.022 [0.015, 0.033]	0.006 [0.004, 0.009]	0.553 [0.487, 0.603]
Public sector outreach (LHW)	0.534 [0.468, 0.613]	0.076 [0.055, 0.097]	0.141 [0.118, 0.174]	0.009 [0.004, 0.015]	0.008 [0.006, 0.011]	0.763 [0.678, 0.810]
Social marketing	1.440 [1.335, 1.516]	0.580 [0.544, 0.626]	0.512 [0.475, 0.550]	0.115 [0.101, 0.127]	0.046 [0.042, 0.050]	2.702 [2.627, 2.810]
Received FP services only in the last 12 months	2.693 [2.690, 2.696]	1.064 [1.062, 1.066]	0.899 [0.898, 0.901]	0.193 [0.192, 0.194]	0.076 [0.075, 0.077]	4.944 [4.940, 4.948]
Total modern method users (including those that received the service/product before the past 12 months)	4.819 [4.816, 4.823]	1.901 [1.899, 1.903]	1.145 [1.144, 1.147]	0.241 [0.241, 0.242]	0.113 [0.112, 0.114]	8.224 [8.220, 8.230]
Traditional users	1.996 [1.994, 1.999]	0.509 [0.507, 0.510]	0.384 [0.382, 0.385]	0.104 [0.103, 0.104]	0.036 [0.036, 0.037]	3.039 [3.036, 3.042]
Total FP users	6.815 [6.810, 6.822]	2.41 [2.406, 2.413]	1.529 [1.526, 1.532]	0.345 [0.344,0.346]	0.149 [0.148, 0.151]	11.263 [11.256, 11.272]

95% CI in parentheses

**Fig. 1:**
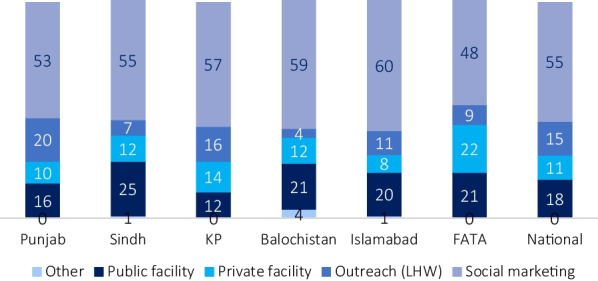
Share of MWRA served in the past year by source (%)

Social marketing, or self-procured contraceptives, account for 55% of women served each year, followed by public facilities (18%) and then LHW outreach (15%) (Fig. 1). Private facilities, including social franchises, serve around 11% of users each year. Within these categories, there are minor variations among provinces; for example, Sindh has the largest share served by public facilities (25%). LHW outreach serves the fewest in Sindh (7%) and Balochistan (4%) and the highest in Punjab (20%). Corresponding users are shown in Table 1.

Table 2 shows current CPR, the targeted rates for CPR as recommended by the CCI, and the estimates of the number of total MWRA that must be served to reach the targets for 2025. To achieve the desired CPR, an estimated 20.02 million MWRA (8.76 million additional users) must be using some form of FP. Thus, the number of MWRA to be served to reach CCI-specified CPR targets must increase by 178% nationwide over the current number of FP users, or the current footprint of service delivery must expand by 1054% if one assumes that LHW outreach and social marketing are maximally utilized and will likely remain unchanged, and therefore that any changes can only come from public or private health facilities.

**Table 2:** MWRA users to be served by CCI targets (in millions)

	Punjab	Sindh	KP	Balochistan	Islamabad	National
Current CPR (2017)	38%	31%	31%	20%	46%	34%
CPR targets for 2025 recommended by CCI	54%	47%	46%	36%	62%	50%
Total MWRA needed to reach 50% CPR in 2025	11.47 [11.46, 11.47]	4.446 [4.443, 4.450]	2.858 [2.856, 2.860]	0.835 [0.834, 0.837]	0.298 [0.297, 0.298]	20.02 [20.02, 20.03]
Additional MWRA needed to reach 50% CPR	4.652 [4.649, 4.656]	2.036 [2.034, 2.039]	1.329 [1.327, 1.331]	0.490 [0.489, 0.491]	0.148 [0.147, 0.149]	8.760 [8.755, 8.765]
Percent increase needed to reach 50% CPR	168%	184%	187%	242%	199%	178%
Percent increase needed if only considering public/private facilities	1245%	865%	830%	1019%	1057%	1054%

95% CI in parentheses

## Discussion

We use national survey data to depict the number of MWRA that must be served to reach national targets for CPR by 2025 and compare it against the current output of the healthcare delivery system. The comparison highlights the rather large gap between the current situation and the ask. More importantly, this framing is distinct from the current debate which revolves around raising CPR by a few percentage points each year.

Pakistan has seen a stagnant CPR for over two decades, despite moderate funding and considerable political support for FP. The major factors contributing to reluctance towards contraceptive use include lack of knowledge, lack of access to healthcare centres, familial restrictions, social constraints and clinical concerns regarding contraception [18]. Identifying the actual number of MWRA to be reached highlights both the immense gap between the current situation and the goal, but also helps identify which types of services, personnel and commodities to make available in which locations. This specificity is necessary since a tenfold increase in the number of MWRA to be served from the existing infrastructure asks vastly different questions than a marginal few percent changes. We discuss some of the options to meet this challenge based on national data.

In Pakistan, much of the advocacy has focused on increasing funding for FP. However, more than half of the government funding remains unutilized, and most public sector funds pay for salaries [19], while health facilities in the public or private sector are highly underutilized [9]. This means that conventional solution levers such as more funds, facilities, personnel or training will do little if funds are underspent and the trained personnel or new facilities are not visited by clients. Fortunately, this also highlights the considerable scope for scaling up services in both the public and private sectors, without requiring additional funds, facilities, personnel or training.

Annual data suggest that public sector outreach (LHW) [20, 21] and social marketing have been expanding very slowly, and may have reached a plateau relative to population [9–12, 22]. Hence, the only two channels where service delivery can expand are either public or private health facilities, which combined currently serve around 1.43 million MWRA each year [9], at an average of less than five FP clients a day (manuscript in preparation). The low utilization with high fixed costs also means that public sector facilities are expensive, at US$ 28 per user served per year, compared to private facilities [14, 23, 24].

To add nine million additional users, these facilities will have to expand their clientele by around tenfold. This increase may happen either spontaneously as clients recognize the need for FP and seek these services out, or they may be guided to the facilities through outreach. Several behavioural change communication programmes have attempted to increase the demand for FP [25, 26]. In national surveys, MWRA are consistently aware of the need for FP and different contraceptive methods available; however, knowledge does not translate consistently into actual use [10]. On the other hand, the use of outreach workers has been very successful in initiating MWRA into FP use and in guiding them to seek contraceptive services from facilities, as seen internationally [27, 28], as well as from Pakistan: the LHW programme [29, 30], Health and Nutrition Development Society (HANDS) MARVI [Marginalized Areas RH (Reproductive Health) and FP Viable Initiatives] workers project [31, 32], the Sukh Initiative [33] and the Akhter Hameed Khan Foundation (AHKF) Aapis Initiative [34]. FP driven by outreach workers often costs less than services through stand-alone facilities [23], and private sector outreach is cheaper than that in the public sector [14, 34–37].

Urban slums account for 30% of the population and are home to nearly half of all poor in Pakistan [38, 39]. These remain largely underserved by public or private programmes including FP [9]. And yet, CPR among urban poor is very similar to rural poor [10]. Given that urban poor are clustered close to each other and near many facilities, they are a large and easy-to-reach underserved population which can be targeted easily and at lower costs. Another important consideration may be to offer a more long-term product mix (such as implants, IUCDs and sterilization) to rural residents, since they are more difficult to reach, while urban residents may be offered a more diverse mix where frequent encounters with the health system will allow certain flexibility [40].

Supply of contraceptives across the country has been inconsistent [41, 42], and it contributes to facility underperformance and lowers client confidence, thereby lowering demand. The inconsistency stems partly from a sporadic lack of funding for products but is mostly due to delays in procurements and underutilization of funds [42, 43]. Some of the procurement issues may be addressed by leveraging the fact that population and FP are considered emergencies. The legal definition of such an emergency may be invoked to allow import of contraceptives from any country to lower tariffs and regulatory barriers to such imports and incentivize local manufacturing of some contraceptives through regulations where feasible.

Finally, although socially marketed supplies may appear to have saturated, this would be true under the assumptions of current demand for FP which depends on how couples perceive the need for more children, their own agency in managing their families, and how these intersect with their access and the ability or willingness to pay for these services. All of these may be modulated by effective behaviour change communication, resulting in increased demand for FP by couples [26], and in turn, leading to considerable expansion in the social marketing channel to meet this demand. There is some evidence that once couples become comfortable with the use of FP, they will continue to use and even pay for it [44]; however, more research is needed to understand the willingness to pay and price points for privately purchased contraception.

Demand creation remains a major gap. At the moment, it appears that while successful outreach and accessibility programmes tend to increase CPR in communities, often such uptake is short-lived and reverts once the programme concludes. In other words, the uptake is driven by supply with insufficient internal demand for contraception for many couples. This may be a contextual issue where the use of contraception will instil long-term usage behaviour [45]. In Pakistan, as in many developing countries, it appears that free supplies for health products such as contraceptives and vaccines are sometimes considered an imposition rather than a benefit and are often discounted in their value compared to commercially available goods. The roles of these conflicting and other issues are unclear in communities and require further research, which should complement and be embedded within current and future programmes. In fact, quick testing of discrete demand creation interventions to learn from the rapid-trial A/B testing paradigm from computers and social media may be made a routine part of programmes [46].

## Recommendations for policy uptake

To reach 50% CPR by 2025, Pakistan will have to increase its quantum of services from public or private facilities by 14-fold. This increase will have to be supported by outreach that refers clients to public or private sector clinics, many of which are underutilized. Community-based, low-cost private sector outreach solutions are more cost-effective than the government’s LHW programme, which is now costly. While much private sector programming has been funded by donors, there are precedents for the public sector contracting of services to the private sector, such as the People’s Primary Healthcare Initiative (PPHI) and other models of contracting out government clinics to private entities [47]. Funding may be allocated to pay for outreach by the private sector, but without the high costs of the public sector. In fact, low-cost initiatives such as the AHKF model in urban settings [34] and the MARVI initiative in rural settings [31, 32] may be considered to benchmark costs of such contracting.

Urban slums are seldom served by public or private sector programmes and thus constitute a large and easy-to-reach yet underserved subgroup of the population. There are few clinics and seldom any outreach services in many urban slums, where population numbers are often underrepresented. On the other hand, distances are shorter. and even if facilities are not in particular neighbourhoods, they can be accessed relatively easily. They are underutilized either because clients do not know when to use them, that is, low demand for them, or because clients do not visit them because they find services and supplies inconsistent or of poor quality. Government can ensure commodity supplies through additional and earmarked funding for contraceptives and by lowering tariff or regulatory barriers to import or local manufacture of contraceptives. Quality may be improved through community feedback mechanisms that allow clinic managers to learn about and respond to client needs [48, 49].

Ultimately, FP in society will have to be client-driven to be sustainable. Some of this demand may be created through programming, such as outreach that highlights the need and means to clients, but more nuanced demand creation that is contextualized in cultural mores is needed through research and testing of discrete interventions within programmes and dedicated research into cultural constructs of FP use.

On the other hand, increased funding, training additional providers and opening new facilities are least likely to help add additional users in the initial phases, where optimization of existing resources is more warranted.

## Limitations

This paper uses survey data from the Pakistan DHS 2017–18 and Population Census-2017 to estimate the number of women that use FP methods; thus the study is subject to the methodological limitations of these surveys, including over- or underrepresentation of certain provinces. The confidence interval may be large for some of the smaller provinces and less common service delivery channels.

## Conclusions

We triangulated nationally available data to arrive at a discrete number of women that must be served to meet national goals. Given the persistent underutilization of funds and existing facilities, it is unlikely that increased funding for FP, more providers or training would help immediately. The most effective policy changes that will likely help include a shift in focus to urban slums, adding outreach (preferably private sector, given the high public sector outreach costs) for support and referral to existing public and private sector facilities, and ensuring that procurement of commodities is prioritized as a means to achieving a tenfold increase in the service delivery footprint of current facilities.

## Data Availability

The study uses request-based available data from the Pakistan Demographic and Health Survey 2017–18 (https://microdata.worldbank.org/index.php/catalog/3411), data from Population Census-2017 (https://www.pbs.gov.pk/content/final-results-census-2017-0), and CCI report (https://pakistan.unfpa.org/sites/default/files/pub-pdf/briefing_note-target_setting_cci_recommendations-2020.pdf).

## Appendix

See Table 3.

**Table 3:** Channels of FP methods currently being used

Serial number	Channels of FP methods	DHS sources of FP methods
1	Public facility	Government hospital Rural health centre Family health clinic/Reproductive Health Services Centers Family welfare centre or Family Welfare Worker Mother–child health centre Basic health unit Lady health visitor Community midwife Other public
2	Private facility	Private/nongovernmental organization hospital/clinic Private doctor Dispenser/compounder Other private Hakim Dai, traditional birth attendant
3	Outreach (LHW)	Lady health worker
4	Social marketing	Pharmacy/medical store Shop Friend/relative

## Abbreviations

CCI: Council of Common Interests
CPR: Contraceptive prevalence rate
FP: Family planning
DHS: Demographic and Health Survey
KP: Khyber Pakhtunkhwa
AJK: Azad Jammu and Kashmir
GB: Gilgit-Baltistan
MWRA: Married women of reproductive age
LHW: Lady health worker
IUCD: Intrauterine contraceptive device
